# Knowledge, Adherence, and Perception of Patients on Maintenance Hemodialysis to Treatment Regimens at a Tertiary Care Hospital in Pakistan

**DOI:** 10.7759/cureus.51341

**Published:** 2023-12-30

**Authors:** Fahad Hussain, Sania Ashraf, Muneeb Arshad, Muhammad Mujtaba ur Rehman, Faheem Shahzad Khan, Muhammad Awais Ahmad, Salamat Ali, Muhammad Ahsan Asif, Ali Munawar, Haseeb Mehmood Qadri

**Affiliations:** 1 Nephrology, Lahore General Hospital, Lahore, PAK; 2 Epidemiology and Biostatistics, University of Health Sciences, Lahore, Lahore, PAK; 3 Medical Education, Akhtar Saeed Medical and Dental College, Lahore, PAK; 4 Internal Medicine, Aziz Bhatti Shaheed Teaching Hospital, Gujrat, PAK; 5 Internal Medicine, Jinnah Hospital, Lahore, PAK; 6 Internal Medicine, Shaikh Zayed Hospital, Lahore, PAK; 7 Surgery, Lahore General Hospital, Lahore, PAK

**Keywords:** perception, adherence, knowledge, hemodialysis (hd), chronic kidney disease (ckd), end-stage renal disease adherence questionnaire (esrd-aq), end stage renal disease (esrd)

## Abstract

Background

The management of end-stage renal disease (ESRD) demands meticulous adherence to treatment regimens, encompassing hemodialysis (HD) sessions, medication protocols, dietary guidelines, and fluid restrictions. The intricate interplay of factors impacting treatment adherence warrants comprehensive exploration, particularly within Pakistan.

Objective

To assess knowledge, adherence, and perception regarding the treatment regimens and their determinants among ESRD patients.

Methodology

Employing a nonprobability, consecutive sampling method, this prospective, cross-sectional study was conducted in July and August 2023 at Lahore General Hospital, Lahore, Pakistan. It exclusively enrolled adult patients with a minimum three-month history of hemodialysis. Thorough demographic data were collected, followed by the meticulous administration of a translated version of the End Stage Renal Disease-Adherence Questionnaire (ESRD-AQ) through face-to-face interviews in the native language. IBM SPSS Statistics for Windows, Version 26 (released 2019; IBM Corp., Armonk, New York, United States) was used to acquire descriptive statistics, as well as Pearson’s and Spearman’s correlations and univariate and multivariate regression analysis.

Results

The study encompassed 119 patients, with a mean age of 43.13 ± 14.99 years. Adherence scores revealed means of 921.83 ± 28.37 for males and 865.18 ± 28.81 for females, out of 1200. Notably, only 10.1% demonstrated good adherence, 31.9% displayed moderate adherence, and 58% exhibited poor adherence. A statistically significant association emerged between better adherence and access to personal transportation (β=-0.225; 95% CI -178.24 to -20.77, p=0.014), with no other demographic factors predicting adherence.

Conclusion

The study underscores the sobering reality of minimal optimal adherence. Chief impediments include anxiety, alongside challenges such as fistula complications, financial constraints, transportation barriers, and inadequate counseling and motivation. Evidently, robust patient education, sustained motivation, and unwavering support from healthcare providers and institutional entities are imperative to surmount the multifaceted barriers that compromise treatment adherence.

## Introduction

When there is greater than 50% anatomical or physiological damage to the kidneys or an estimated glomerular filtration rate (eGFR) that is less than 60 ml/min/1.73 m2 that lasts for at least three months, the condition is referred to as chronic kidney disease (CKD) [1]. CKD is a significant global health issue that affects more than 109.9 million individuals in developed countries and 387.5 million people in the developing world. This number is expected to rise further in the coming years [2]. Worldwide, it contributed to 956,000 fatalities in 2013, up from 409,000 in 1990 [3]. According to the 2017 Global Burden of Disease research, renal disorders cause 1.2 million fatalities globally, and the all-age mortality rate linked to CKD increased by 41.5% from 1990 to 2017 [4]. Regarding the eGFR level, patients with CKD go through several stages of the disease, labeled from one to five [1]. The last stage of CKD, known as end-stage renal disease (ESRD), is characterized by an irreversible decline in kidney function, necessitating either a renal transplant or a continuing regimen of long-term hemodialysis to maintain life [1]. The increasing prevalence of diabetes and hypertension in Pakistan makes CKD a prevalent health problem here [5]. There are more than 100 newly diagnosed cases of ESRD annually per million people, and the prevalence is estimated to range from 16.6% to 25% in Pakistan [4].

Hemodialysis (HD) is the preferred and most widely used treatment modality in patients with end-stage renal disease (ESRD) [3,6]. Patients’ strict adherence to the HD sessions attendance, prescription of medications, and dietary and fluid restrictions is crucial to the success of the treatment. Many studies have highlighted that non-adherence to any of these parameters can have negative consequences on the quality of life as well as higher morbidity, mortality, and medical expense rates in these patients [1,7,8,9]. Adherence or compliance is the measure of a patient’s behavior to follow the advice or treatment given by the doctor [10]. Chronic patients, particularly those on HD, frequently struggle with non-compliance with the prescribed treatment plan. The causes of non-adherence include cultural norms, socioeconomic position, health beliefs, treatment attitudes, perception, and awareness of the therapy plan [1,10,11]. There have been few studies regarding adherence among HD patients in Pakistan. A study in Karachi found that 13.4% of patients attended hemodialysis sessions irregularly, while 18% of patients discontinued their hemodialysis treatment [12]. Shehryar Raashid et al. used the End Stage Renal Disease-Adherence Questionnaire (ESRD-AQ) to assess adherence among patients at a hospital in Rawalpindi [4].

The study aimed to explore the intricate challenges surrounding adherence among ESRD patients in Pakistan. It sought to uncover the multifaceted factors impacting adherence, including cost-related barriers, limited healthcare access, and the influence of diverse risk factors. By addressing this critical research gap, the study intends to provide insights for tailored interventions, enhancements to healthcare infrastructure, and the establishment of support systems. These efforts aimed to empower ESRD patients to more effectively adhere to their treatment regimens and, in turn, improve their overall health outcomes.

## Materials and methods

Ethical approval

All procedures performed in this study involving human participants were in accordance with the ethical standards of the Institutional Review Board of Lahore General Hospital, Lahore, with the approval number 0041/2023, dated July 13, 2023, and with the 1964 Helsinki Declaration and its later amendments or comparable ethical standards.

Study design

This prospective, cross-sectional study was carried out from July 9, 2023, till August 10, 2023, at the Primary Hemodialysis Unit (PHU) of Lahore General Hospital, Lahore, which is a 30-bedded facility and works for emergency and elective cases throughout the year.

Inclusion criteria

All elective patients who were conscious with a Glasgow Coma Scale (GCS) of 15/15, had been receiving HD for at least three months, underwent dialysis at least twice per week for an average of three hours, and whose medical records contained complete demographic, clinical, and biochemical data were included in the study.

Exclusion criteria

Emergency cases and patients who did not consent or were on hemodialysis for less than three months were excluded from the study.

Informed consent

Informed consent was obtained from all individual participants included in the study, explaining the nature, purpose, and implications of the study.

Sampling technique and sample size calculation

A nonprobability, consecutive sampling technique was used to recruit the willing participants according to the above-mentioned criteria. The sample size of 119 for this study was calculated using the World Health Organization OpenEpi Calculator by taking a local study as a reference, maintaining the power of the study at 80%, a confidence level of 95%, a margin of error of 5%, and the frequency of chronic kidney disease (CKD) at 8.4% [13,14].

Data collection

All PHU patients were approached and interviewed to fill out the questionnaire, and to accomplish this, our co-authors spent four consecutive weeks collecting data at the hemodialysis facility. The End Stage Renal Disease-Adherence Questionnaire (ESRD-AQ), a standard tool, was employed in this study to evaluate: (1) the level of adherence; (2) perception; and (3) counseling of the patients towards the HD treatment regimen [15]. In addition to conducting the interviews, we gathered clinical and biochemical data from the patient’s medical records. To assess clinical adherence, pre-dialytic serum potassium and phosphate levels, together with interdialytic body weight (IDW), were utilized as clinical markers for compliance with diet, medications, and fluid restriction, respectively. The average of the last three measurements taken within the previous month was used for all biochemical indicators. To determine the IDW, the post-HD weight was subtracted from the pre-HD weight (pre-HD weight minus post-HD weight), which represents the amount of fluid consumed between dialysis sessions. As a biochemical indicator of fluid restriction adherence, the mean of three consecutive IDW measurements was employed.

Data analysis

Using IBM SPSS Statistics for Windows, Version 26 (released in 2019; IBM Corp., Armonk, New York, United States), basic frequencies and descriptive statistics were calculated. Pearson’s and Spearman’s correlations were also calculated in addition to the univariate and multivariate regression analyses. The p-value significance level was set at <0.05. By adding the answers to six specific questions (numbers 14, 17, 18, 26, 31, and 46 from the ESRD-AQ), the overall adherence behavior score is calculated. The highest total score for these questions is 1200, and higher values indicate better compliance. This score was categorized into poor (<700), moderate (700-999), and good (1000-1200). The sum of questions 11, 22, 32, and 41 was used to calculate the importance of adherence to different treatment modalities as perceived by the patients. All these questions had five possible responses, and they were categorized into three categories: little/not important, moderately important, and highly/very important.

The co-authors, whose native language is Urdu, translated the ESRD-AQ scale into Urdu for this study and thoroughly compared it to the original English to maintain its original meanings. A pilot study was conducted involving 10 participants to validate the translated version. The ESRD-translated AQ's version in Urdu is available with the corresponding author upon request.

## Results

A total of 119 patients met the inclusion criteria and were recruited for the study. The patients had a mean age of 43.13 ± 14.99 years, in a range of 17-85 years. Thirty-five (29.4%) patients had no chronic diseases as a known cause of ESRD. Only one (1) patient (0.8%) had a previous kidney transplant. Two (1.7%) of the patients had peritoneal dialysis in the past. Approximately half (49.6%) of the patients used their transportation to reach the dialysis center, and the majority (82.4%) attended the dialysis sessions along with their family. Most of the patients (78.2%) described their dialysis schedule as convenient. Table 1 shows the selected socio-demographic and clinical characteristics of the study sample.

**Table 1:** Socio-demographic and clinical characteristics of the studied sample, in terms of number (n) and percentage distribution (%) HD: hemodialysis

Variable	Number of patients, n and their percentage distribution, %
Age (mean ± SD)	43.13 ± 14.99
Gender	
Male	63 (52.9%)
Female	56 (47.1%)
Diabetes mellitus	
Yes	8 (6.7%)
No	111 (93.3)
Hypertension	
Yes	43 (36.1%)
No	76 (63.9%)
Diabetes mellitus and hypertension	
Yes	33 (27.7%)
No	86 (72.3%)
Renal transplant	
Yes	1 (0.8%)
No	118 (99.2%)
Peritoneal dialysis	
Yes	2 (1.7%)
No	117 (98.3%)
How the patient reached the HD center	
Personal transport	59 (49.6%)
Public transport/others	60 (50.4%)
Accompanied by family to HD	
Yes	98 (82.4 %)
No	21 (17.6%)

Adherence to the four treatment modalities was assessed and is shown below in Table 2. Adherence to HD sessions was the highest, with an average score of 246.64 ± 94.49 out of a maximum score of 300.

**Table 2:** Mean and standard deviation (SD) of ESRD-AQ adherence scores for different parameters ESRD-AQ: End Stage Renal Disease-Adherence Questionnaire; HD: hemodialysis

ESRD-AQ item #	Adherence	Range of score	Mean score ± SD
14	HD- attendance	0-300	246.64 ± 94.49
17	The episode of shortening HD	0-200	142.86 ± 62.53
18	Duration of HD shortening if shortened	0-100	49.37 ± 47.89
26	Adherence to medication	0-200	166.21 ± 54.57
31	Adherence to fluid restrictions	0-200	149.58 ± 60.19
46	Adherence to dietary restrictions	0-200	139.08 ± 50.49

The overall adherence behavior of each patient was assessed by summing the scores of questions 14, 17, 18, 26, 31, and 46 (Table 3).

**Table 3:** Overall ESRD-AQ adherence score categories, in terms of frequency of occurrence (n) and percentages (%) ESRD-AQ: End Stage Renal Disease-Adherence Questionnaire

Adherence category	Total score	Frequency, n	Percentage, %
Poor	<700	69	58%
Moderate	700-999	38	31.9%
Good	1000-1200	12	10.1%

**Figure 1:**
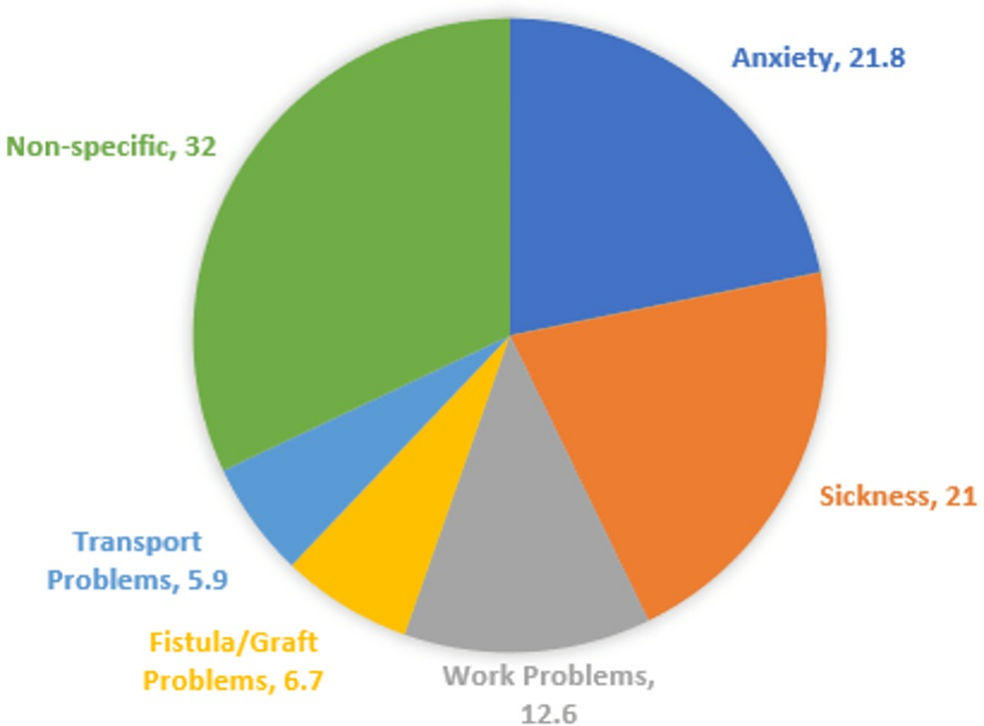
Different causes of non-adherence to treatment regimens, in terms of percentages (%)

The most common cause of non-adherence was anxiety as shown in Figure 1.

There was no statistically significant correlation between adherence behavior and age (Spearman correlation: p=0.129, r=0.140) or the number of years since starting (Spearman correlation: p=0.779, r=0.026) or gender (male=921.83 ± 28.37; female=865.18 ± 28.81; p=0.641, r=0.043). The mean ± SD for pre-HD serum potassium of studied patients was 4.70 ± 0.70 mmol/l, while the median (Q1-Q3=4.2-5.1) was 4.50 mmol/l. There was no significant correlation between pre-dialytic serum potassium level and diet adherence score (Spearman correlation: p=0.755; r=− 0.029). The mean IDW was 2.48 ± 0.80 kg, while the median (Q1-Q3=2-3) was 2.0 kg. There was no significant correlation between IDW and adherence to the fluid restriction (Spearman correlation: p=0.13, r=− 0.14). The mean pre-dialytic phosphate level was 4.52 ± 1.70 mg/dl, while the median (Q1-Q3=3.8-5.0) was 4.30 mg/dl. The levels of serum phosphate are within normal levels, indicating the inadequacy of compliance. There was no significant correlation between medication adherence and pre-dialytic phosphate level (Spearman correlation: p=0.801, r=0.023). A significant negative correlation was found between IDW and the adherence behavior score (p=0.003, r=-0.027). All the correlations are depicted in Table 4, and the significant values are marked with an asterisk (*).

**Table 4:** Correlational analysis of clinical parameters and adherence scores using Pearson's and Spearman's correlation IDW: interdialytic body weight; HD: hemodialysis

Variables	Gender	Pre-dialysis phosphate levels	Pre-dialysis potassium levels	Interdialytic weight	HD adherence score	Medication adherence score	Fluid adherence score	Diet adherence score	Overall adherence score
Age	r : 0.043	r : 0.023	r : -0.071	r : 0.060	r : 0.114	r : 0.007	r : 0.182*	r : 0.034	r : 0.140
	p : 0.641	p : 0.805	p : 0.444	p : 0.518	p : 0.217	p : 0.940	p : 0.048*	p : 0.717	p : 0.129
Gender	-	r : 0.045	r : -0.154	r : 0.084	r : -0.026	r : 0.032	r : -0.068	r : -0.075	r : -0.162
	-	p : 0.63	p : 0.094	p : 0.363	p : 0.775	p : 0.734	p : -0.461	p : 0.419	p : 0.079
Pre-dialysis phosphate levels	-	-	r : 0.093	r : 0.175	r : 0.122	r : 0.023	r : 0.134	r : 0.055	r : 0.104
	-	-	p : 0.316	p : 0.057	p : 0.186	p : 0.801	p : 0.146	p : 0.555	p : 0.259
Pre-dialysis potassium levels	-	-	-	r : 0.162	r : -0.139	r : -0.096	r : -0.058	r : -0.029	r : -0.034
	-	-	-	p : 0.079	p : 0.133	p : 0.301	p : 0.533	p : 0.755	p : 0.716
IDW	-	-	-	-	r : -0.311*	r : -0.093	r : -0.140	r : -0.095	r : -0.0270*
	-	-	-	-	p : 0.001*	p : 0.315	p : 0.130	p : 0.302	p : 0.003*
HD adherence score	-	-	-	-	-	r : 0.295*	r : 0.119	r : 0.338*	r : 0.671*
	-	-	-	-	-	p : 0.001*	p : 0.199	p : 0.000*	p : 0.000*
Medication adherence score	-	-	-	-	-	-	r : 0.208*	r : 0.207*	r : 0.507*
	-	-	-	-	-	-	p : 0.023*	p : 0.024*	p : 0.000*
Fluid adherence score	-	-	-	-	-	-	-	r : 0.324*	r : 0.438*
	-	-	-	-	-	-	-	p : 0.000*	p : 0.000*
Diet adherence score	-	-	-	-	-	-	-	-	r : 0.521*
	-	-	-		-	-	-	-	p : 0.000*

We assessed the perceptions of the studied patients towards various HD treatment modalities (Table 5). Perception towards medication adherence had the highest score, with 85.7% of studied patients believing that following the dialysis schedule was highly or very important. Perception towards fluid restriction was the lowest, with 68.1% of studied patients believing that it was highly or very important to watch the amount of fluid taken daily. The sum of perception scores obtained by summing questions 11, 22, 32, and 41 yielded a median of 6 (Q1-Q3=5-8) and a mean of 6.88 ± 2.63.

**Table 5:** Patients' perception of adherence to different treatment regimens in terms of frequency (n) and percentage distribution (%) ESRD-AQ: End Stage Renal Disease-Adherence Questionnaire

ESRD-AQ item #	Perception on importance	Highly/very important, n (%)	Moderately important, n (%)	Little/no important, n (%)
11	How important do you think it is to follow your dialysis schedule?	100 (84.0)	9 (7.6)	10 (8.4)
22	How important do you think it is to take your medicines as scheduled?	102 (85.7)	14 (11.8)	3 (2.5)
32	How important do you think it is to limit your fluid intake?	81 (68.1)	23 (19.3)	15 (12.6)
41	How important do you think it is important for you to watch your diet daily?	92 (77.3)	17 (14.3)	10 (8.4)

Eight items in the ESRD-AQ discuss the counseling received by patients for various treatment modalities. For each treatment modality, the patients were asked two questions about counseling. The most negative answer was "never." Table 6 shows the percentages.

**Table 6:** Recent analysis of patients having counseling regarding the importance of different treatment regimens for ESRD, in terms of percentage distribution (%) ESRD-AQ: End Stage Renal Disease-Adherence Questionnaire

ESRD-AQ item #	Counseled within the last month (%)	Never counseled (%)
9	92.4	0
10	89.1	0.8
20	89.1	0
21	76.5	0
29	69.7	6.7
30	68.9	4.2
39	62.2	10.9
40	69.7	3.4

The univariate and multivariate analyses predicted that the patients having their personal transports were more likely to have better adherence scores. No other parameter could predict a significant change in the adherence score. Table 7 shows this analysis, and significant values are marked in an asterisk (*).

**Table 7:** Regression analysis of the factors associated with overall adherence, with a confidence interval set at 95% and a p value of 0.05

Parameters	Univariate analysis				Multivariate analysis			
	Unstandardized coefficients (B)	Standardized coefficient (Beta)	p-value	95% confidence interval	Unstandardized coefficients (B)	Standardized coefficient (Beta)	p-value	95% confidence interval
Age	-	-	-	-	0.588	0.040	0.336	-0.62 to 1.79
Gender	-56.647	-0.128	0.165	-136.93 to 23.64	-	-	-	-
Availability of the attendance	-34.609	-0.060	0.518	-140.41 to 71.19	-	-	-	-
Transport used	-99.51	-0.225	0.014*	-178.24 to -20.77	-	-	-	-

## Discussion

In Pakistan, where healthcare is a luxury rather than a fundamental right, access to dialysis therapy is limited due to high costs and a lack of availability [16]. However, a significant majority of patients undergoing HD in Pakistan struggle to adhere to their doctor's recommendations about nutrition, fluid intake, and dialysis [11]. Hence, this study analyzed the perception and adherence behavior using the ESRD-AQ among the patients on maintenance hemodialysis at our center.

Hypertension and diabetes are found to be the most common preventable risk factors for CKD patients in Pakistan [16]. Naalweh et al. noted that 55% of the patients had hypertension, and 39% had diabetes [6]. A study in Saudi Arabia showed that the majority of HD patients had hypertension as their diagnosis and more than one-third were diagnosed as diabetics. Similarly, an Indian study found that 24% of HD patients had diabetes and 81% had hypertension [6]. While our study had 6.7% of patients with diabetes mellitus, 36.1% were hypertensive, and 27.7% had both.

The overall adherence scores showed that only 12 (10.1%) out of 119 patients showed good adherence scores, while 38 (31.9%) and 69 (58%) patients showed moderate and poor adherence, respectively. In contrast, Naalweh et al. noted good adherence scores in 55.5% of the patients using the same questionnaire [6]. Various types of research have found that younger age, educational status, daily life activity disturbance, inconvenient schedule, pain and sense of discomfort with arteriovenous (AV) fistula, employment status, and psychological burden are some of the causes of poor adherence among ESRD patients [8,11,17-20]. Similarly, our study listed anxiety, employment issues, sickness like fistula or graft problems, and transport as the major causes of non-adherence (Figure 1).

Adherence rates to attend HD sessions on time varied from 65% to 100% among different studies [6,8,19,20]. One study showed that the rate of HD session adherence was 33.6% [18]. However, the methods used to determine these rates differed across the studies analyzed. In our study, the ESRD-AQ-based HD session adherence score of 246.64 ± 94.49 was comparable to the score of 259.90 ± 75.84 in a study from Rawalpindi [4] and much lower than the score of 296.36 ± 26.78 in the study conducted by Naalweh et al. in Palestine [6]. Additionally, in our study, 21.8% of patients considered their dialysis schedule to be inconvenient, and 13.45% of patients said that their work was affected by their dialysis schedule. About 21.8% of the patients labeled anxiety as the main reason to miss dialysis sessions. Around 57.9% of the patients discontinued their dialysis sessions because of some physical discomfort or sickness. Only 5.9% of the total patients listed their transport as the problem for missing dialysis sessions. However, transport was the only indicator to exhibit significance in the regression analysis, depicting that people using their transport to reach the dialysis center were more likely to show better adherence.

Adherence to medication intake ranged from 19% to 98.8% in different studies [4,6,8,18]. Nearly 58% of the patients were completely compliant with the medication, and the most common reason to miss the medication, highlighted by 21% of the patients, was the medication cost. The mean ESRD-AQ medication adherence score in other studies was 184.32 ± 37.83 and 182.67 ± 32.74 [4,6]. However, our study noted a much lower (166.21 ± 54.57) medication adherence score.

Overall, adherence rates to fluid recommendations varied from as low as zero percent to as high as 96.6% [4,6,8,10,18,20]. Halle et al. quote a high prevalence (64%) of non-adherence to fluid restriction among patients undergoing two weekly HD sessions despite good knowledge in Pakistan [20], while this percentage was 24 in our scientific study. The mean ESRD-AQ fluid restriction score was 134.16 ± 54.28 and 140.68 ± 53.78 [4,6]. Our study noted a slightly better (149.58 ± 60.19) fluid restriction score. Perfect compliance with the fluid restriction was noted in 43.7% of the patients, and 18.6% mentioned a lack of understanding as the main reason for non-adherence to the fluid restriction.

The overall compliance rate with dietary restrictions ranges from 98.8% to 8.9% [4,6,8,10,18,20]. We also found that only 26.6% of our participants were completely adherent to the diet recommendations. However, the inability to control themselves despite knowing was the most common (33.6%) reason for non-adherence in other patients. In other studies, the mean ESRD-AQ fluid restriction score was 148.02 ± 46.33 and 134.55 ± 52.01 [4,6]. Our study also documented comparable scores (139.08 ± 50.49) in this regard.

Lack of renal health-related knowledge and counseling is the most important modifiable factor of all the factors noted [17]. An overburdened healthcare setup might also lead to non-adherence due to the unavailability of proper counseling time for patients [17]. However, in a study, 44.5% of the patients claimed that diet counseling from health professionals was rarely provided. In addition, the quality of patient nutrition education is also likely to be undermined by nutrition knowledge incompetency among healthcare professionals [21]. Lack of dietetic services in the HD setting is a critical problem also reported in Malaysia and other Asian countries. As such, nephrologists and nurses have become the primary sources of dietary information for HD patients [21]. Although more than 75% of the patients in our study admitted that they were counseled about all four aspects of the HD regimen, the main source of the counseling were the nurses and doctors of the center. This stresses the importance of having a multidisciplinary approach with a professional counselor and a dietitian for proper information delivery to HD patients. Another study suggested that the patient education program and nurse-led telephone follow-up improve treatment adherence in the four dimensions of HD attendance, medication use, fluid restrictions, diet recommendations in HD patients, and clinical parameters [1].

Limitations

Numerous issues with the aforementioned study need to be addressed thoroughly to improve the results. In the healthcare system under review, access to healthcare services is seen more as a luxury than a fundamental human right. As a result, the patients receive inadequate care. Patients exhibit poor adherence and compliance to their therapy as a result of their lack of mental and physical preparation before their treatment process, which completely changed the findings of our study. Patients and staff both lack fundamental knowledge. Healthcare professionals who are overworked lack compassion for the pain of others, which also affects how patients respond. The study was carried out in a public setting at a tertiary care hospital where the majority of guests are in dire financial straits. If it had been done in a private setting, the results might have been different because patient compliance, tension, and anxiety are often greatly impacted by financial independence.

Clinical recommendations and future implications

Our study revealed that worry, illness, work challenges, fistula/graft issues, and transportation problems to the dialysis centers were the main causes of non-adherence. Before scheduling a patient for any treatment that will have a long-term impact on his or her life, all of these factors should be properly taken into account. Before the procedure, thorough counseling sessions should be conducted, covering all facets of the disease, treatment options, and potential repercussions from untreated disease. The majority of these patients indicated how their sickness and treatment were interfering with their everyday activities and reputation at work, so it is important that they receive adequate support from the workplace. Healthcare providers should teach patients how to properly care for a fistula or graft.

The analysis identified transportation as the most significant demographic component. Compared to those who used public transportation, individuals who used their vehicles showed better adherence to their treatment. Their response to the disease can be significantly altered positively only by offering the sick individual appropriate transportation amenities.

The most significant modifiable element to affect a patient's response is their level of awareness and counseling regarding renal health. To prevent these issues, the general public has to be educated about how to maintain optimal renal health. Another significant factor in the patient's subpar reaction to his illness and treatment is an overburdened healthcare system. It is necessary to upgrade healthcare facilities. People are ignorant of their illnesses and the course of their treatments. Thus, educating the public is a duty of the healthcare system.

Patients receiving hemodialysis often fail to adhere to the necessary dietary and fluid restrictions due to inadequate patient counseling. Every dialysis facility needs to have a nutritionist and a counselor for the benefit of the patients, as well as to lessen the workload of the doctors and nurses who serve as the patients' only counselors and dietitians. Above all, a patient receiving hemodialysis should have a multidisciplinary approach employed to address all facets of the procedure. The results of patient understanding, adherence, and compliance with their medication will undoubtedly be improved by patient education programs as well as by nurse-led follow-up sessions with patients.

## Conclusions

The study exclusively concludes that there is poor compliance towards all major factors. There is no significant correlation between age, gender and other commodities but education plays some role in adherence to medication and diet regimen. The most common causes of non-adherence found in our study are, anxiety, sickness, work problems, fistula and graft problems and some included transport problems. Most of the patients were adherent to the medication regimen and their perception towards medication adherence had the highest score.

Overall only a few patients (10.1%) had good adherence. Anxiety was the most common problem for non-adherence. However, fistula problems, financial issues, transport problems, and lack of counseling and motivation were some of the other reasons identified. The patients that showed moderate adherence were mostly non-compliant in fluid restriction and diet restriction and 31.9% of patients showed moderate adherence while 58% showed poor adherence.
